# Hedgehog target genes regulate lipid metabolism to drive basal cell carcinoma and medulloblastoma

**DOI:** 10.21203/rs.3.rs-3058335/v1

**Published:** 2023-08-03

**Authors:** Vikas Daggubati, Akshara Vykunta, Abrar Choudhury, Zulekha Qadeer, Kanish Mirchia, Olivier Saulnier, Naomi Zakimi, Kelly Hines, Michael Paul, Linyu Wang, Natalia Jura, Libin Xu, Jeremy Reiter, Michael Taylor, William Weiss, David Raleigh

**Affiliations:** University of California San Francisco; University of California San Francisco; University of California, San Francisco; University of California San Francisco; University of California San Francisco; The Hospital for Sick Children; University of California San Francisco; University of Washington; University of California, San Francisco; University of California San Francisco; University of California, San Francisco; University of Washington; Department of Biochemistry and Biophysics, Cardiovascular Research Institute, University of California San Francisco, San Francisco, CA; University of Toronto; University of California San Francisco; University of California San Francisco

## Abstract

Hedgehog (Hh) signaling is essential for development, homeostasis, and regeneration^1^. Misactivation of the Hh pathway underlies medulloblastoma, the most common malignant brain tumor in children, and basal cell carcinoma (BCC), the most common cancer in the United States^2^. Primary cilia regulate Hh signal transduction^3^, but target genes that drive cell fate decisions in response to ciliary ligands or oncogenic Hh signaling are incompletely understood. Here we define the Hh gene expression program using RNA sequencing of cultured cells treated with ciliary ligands, BCCs from humans, and Hh-associated medulloblastomas from humans and mice (Fig. 1a). To validate our results, we integrate lipidomic mass spectrometry and bacterial metabolite labeling of free sterols with genetic and pharmacologic approaches in cells and mice. Our results reveal novel Hh target genes such as the oxysterol synthase *Hsd11β1* and the adipokine *Retnla* that regulate lipid metabolism to drive cell fate decisions in response to Hh pathway activation. These data provide insights into cellular mechanisms underlying ciliary and oncogenic Hh signaling and elucidate targets to treat Hh-associated cancers.

Hh ligands such as Sonic Hedgehog (Shh), Desert Hedgehog (Dhh), and Indian Hedgehog (Ihh) are tissue-specific lipoproteins that bind to Patched1 (Ptch1), remove Ptch1 from cilia, and allow Smoothened (Smo) to accumulate in cilia to activate the Gli family of transcription factors that regulate Hh target gene expression^4–6^. Sterol and oxysterol lipids underlie Ptch1 regulation of Smo^7–9^ and Smo activation^10–14^. Lipidomic mass spectrometry reveals cilia-associated lipids such as 24(*S*),25-epoxycholesterol (24(*S*),25-EC) and 7β,27-dihydroxycholesterol (7β,27-DHC) bind to Smo, recruit Smo to cilia, and activate the Hh pathway^12^. Free sterol labeling using bacterial metabolites shows Hh signaling regulates the lipid microenvironment of cilia^15^. Synthetic small molecules such as 20(S)-hydroxycholesterol (20-OHC) and Smo Agonist (SAG) also bind to Smo, recruit Smo to cilia, and activate the Hh pathway. Nevertheless, it is unknown if the gene expression programs underlying cellular responses to Hh ligands, Smo-activating oxysterols, and SAG are convergent or divergent. Moreover, it is unknown if ciliary ligands and oncogenic Hh signaling regulate overlapping or non-overlapping gene expression programs.

In the absence of a granular understanding of Hh target genes, QPCR for *Gli1* and *Ptch1*, or Gli luciferase reporters are used as surrogate readouts for the Hh gene expression program. Only a handful of additional genes, such as *Bcl-2, N-myc, Acbg2, Fgf4, Cdk6,* and *Vegfa* have been implicated as direct targets of the Hh gene expression program, but the available data suggest that these genes are unlikely to account for the diversity of cell fate decisions that are controlled the Hh pathway^16–20^. Hh ligands (Shh, Ihh), Smo-activating oxysterols (24(S),25-EC, 7β,27-DHC, 20-OHC), and SAG accumulate Smo in cilia (Fig. 1b and Extended Data Fig. 1a) and induce canonical Hh target gene expression (*Gli1, Ptch1*) in NIH3T3 fibroblasts (Fig. 1b and Extended Data Fig. 1b, c). To test the specificity of ciliary ligands for regulating Hh target gene expression, CRISPR/Cas9 genome editing was used to delete *Smo* in NIH3T3 cells (Fig. 1c). *Smo* deletion did not alter ciliary architecture (Extended Data Fig. 2a, b) but blocked Smo accumulation in cilia and blocked canonical Hh target gene expression in response to Hh ligands, Smo-activating oxysterols, and SAG (Fig. 1d–f). RNA sequencing was performed on triplicate NIH3T3 wildtype or *Smo*^−/−^ cultures after treatment with Shh, Dhh, Ihh, 24(*S*),25-EC, 7β,27-DHC, 20-OHC, or SAG (Supplementary Table 1, 2). Differential expression analysis was performed in comparison to RNA sequencing of matched cell lines after treatment with triplicate vehicle controls (BSA for Hh ligands, EtOH for Smo-activating oxysterols, DMSO for SAG). Non-specific gene expression changes were identified and filtered using RNA sequencing of NIH3T3 *Smo*^−/−^ cells treated with the same ligands or vehicle controls, or RNA sequencing of triplicate NIH3T3 wildtype and *Smo*^−/−^ cultures after treatment with 7α,27-dihydroxycholesterol (7α,27-DHC), an oxysterol that does not bind to Smo or activate the Hh pathway^11^ (Extended Data Fig. 3a, b). These analyses revealed a core Hh gene expression program comprised of 143 protein coding genes that were associated with lipid synthesis (*Hsd11β1, Retnla, Hmgcr*), metabolism (*Fdps, Fdft1, Atp6v0a4*), cell signaling (*Gli1, Ptch1*), cell adhesion (*Cldn1*), or angiogenesis (*Edn2, Bdkrb2*) (Fig. 1g, h and Supplemental Table 3).

The Hh gene expression program is well studied in medulloblastoma^21–23^, but Hh target genes in newly diagnosed human BCC are incompletely understood. To address this limitation in our understanding of oncogenic Hh signaling, RNA sequencing was performed on matched tumor and adjacent skin samples from 10 patients with newly diagnosed BCC. Differential expression and ontology analyses demonstrated alterations in cell development, lipid metabolism, and diverse biosynthetic pathways in BCC compared to adjacent skin (Fig. 2a, b and Supplementary Table 4). Lipidomic mass spectrometry on the same 10 pairs of matched BCC and adjacent skin samples showed enrichment of cilia-associated and Smo-activating sterols and oxysterols in BCC compared to adjacent skin, including cholesterol, desmosterol, 7-keto,27-hydroxycholesterol (7k,27-OHC), 24k-cholesterol (24k-C), and 24(S),25-EC^10^ (Fig. 3a). 7-keto-cholesterol (7k-C), a precursor of 7k,27-OHC and 7β,27-DHC that can be catabolized to 7-hydroxycholesterol (7-OHC) by the oxysterol synthase Hsd11β1^24^, was also enriched in BCC compared to adjacent skin, suggesting that the Hh target gene *Hsd11β1* from NIH3T3 cells (Fig. 1g, h and Supplemental Table 3) may be suppressed in BCC. In support of this hypothesis, comparison of differentially expressed genes in human BCC (Fig. 2 and Supplementary Table 4) to differentially expressed Hh target genes in NIH3T3 cells (Fig. 1 and Supplementary Table 3) revealed *Hsd11β1* was suppressed but canonical Hh target genes (*Gli1, Ptch1*) were enriched in human BCC (Fig. 3b).

These data suggest that Hsd11β1 regulates lipid metabolism to inhibit Hh signal transduction, and that Hsd11β1 suppression in BCC facilitates oncogenic Hh signaling to drive cancer cell proliferation. To test this hypothesis, NIH3T3 cells stably expressing dCas9-KRAB CRISPR interference (CRISPRi) machinery^25^ were transduced with sgRNAs targeting *Hsd11β1* (*sgHsd11β1*) or non-targeted control sgRNAs (sgNTC). *Hsd11β1* suppression did not influence Smo accumulation in cilia but enhanced canonical Hh target gene expression in NIH3T3^dCas9 – KRAB^ cells compared to sgNTC (Fig. 3c–e). NIH3T3 cells stably expressing a doxycycline-inducible FLAG-Hsd11β1 construct showed no change in ciliary Smo accumulation with Hsd11β1 over-expression, but Hsd11β1 over-expression attenuated canonical Hh target gene expression in NIH3T3^dCas9 – KRAB^ cells compared to vehicle control (Fig. 3f–h). Hsd11β1 over-expression in ASZ BCC cells suppressed Smo-activating sterols as measured using perfringolysin O (PFO*), a bacterial metabolite that labels free sterol lipids in live cells^15^ (Fig. 3i, j). Hsd11β1 did not localize to ASZ cilia (Fig. 3k), but over-expression of Hsd11β1 attenuated clonogenic growth of BCC cells (Fig. 3l). Hsd11β2 opposes Hsd11β1 and activates the Hh pathway by converting 7-hydroxycholesterol (7-OHC) to 7k-C^24^, and carbenoxolone (CNX), a naturally occurring small molecule that blocks Hh-associated medulloblastoma^12,26^, inhibits Hsd11β2. In ASZ BCC cells, CNX attenuated clonogenic growth and synergized with Hsd11β1 over-expression (Fig. 3l). These data demonstrate Hsd11β1 regulates Smo-activating lipid metabolism to inhibit Hh signal transduction and block cell proliferation *in vitro*.

Cilia-associated sterol and oxysterol lipids drive Hh-associated medulloblastoma growth and response to treatment^12,26^. To identify Hh target genes underlying these phenotypes, differentially expressed Hh target genes from NIH3T3 cells (Fig. 1 and Supplementary Table 3) were compared to differentially expressed genes from (1) RNA sequencing of 250 Hh-associated medulloblastomas compared to 208 Group 3 medulloblastomas from humans^21–23^ (Fig. 4a and Supplementary Table 5), or (2) RNA sequencing of *Math1-Cre SmoM2*^*c*^ genetically engineered mouse models of Hh-associated medulloblastoma (n = 3) compared to age-matched control cerebella from *SmoM2*^*c*^ mice (n = 3)^17^ (Fig. 4b and Supplementary Table 6). *Hsd11β1* was enriched in Hh-associated medulloblastomas compared to Group 3 medulloblastomas from humans (Fig. 4a). *Hsd11β1* was suppressed in *Math1-Cre SmoM2*^*c*^ Hh-associated medulloblastomas compared to control cerebella from mice (Fig. 4b), as was the case for *Hsd11β1* expression in human BCCs compared to adjacent skin (Fig. 2 and Supplementary Table 4). These data suggest Hh target are enriched in Hh-associated cancers compared to related cancers arising in similar anatomic locations, but that Hh target genes that inhibit oncogenic Hh signaling, like *Hsd11β1*, are suppressed in Hh-associated cancers compared to adjacent tissues, like the skin or cerebellum.

*Retnla*, an adipokine that regulates sterol synthase expression to control lipid metabolism^27^, was enriched in NIH3T3 cells after treatment with ciliary ligands (Fig. 1g, h and Supplemental Table 3) and was also enriched in *Math1-Cre SmoM2*^*c*^ Hh-associated medulloblastomas compared to age-matched control cerebella^17^ (Fig. 4b and Supplementary Table 6). To determine if *Retnla* regulates the Hh pathway, NIH3T3^dCas9 – KRAB^ cells were transduced with sgRNAs targeting *Retnla* (*sgRetnla*) or sgNTCs. *Retnla* suppression decreased Smo accumulation in cilia and inhibited expression of canonical Hh target genes in NIH3T3^dCas9 – KRAB^ cells compared to sgNTC (Fig. 4c–e). Moreover, *Retnla* suppression reduced the expression of *Hsd11β2* (Fig. 4f), an oxysterol synthase and positive regulator of the Hh pathway that produces Smo-activating lipids^12^. In support of these data, PFO* labeling was reduced in NIH3T3^dCas9 – KRAB^ cells expressing *sgRetnla* compared to sgNTC (Fig. 4g). NIH3T3 cells stably expressing a doxycycline-inducible FLAG-Retnla construct showed increased ciliary Smo accumulation with Retnla overexpression and increased canonical Hh target gene expression compared to vehicle control (Fig. 4h–j). Retnla overexpression increased *Hsd11β2* expression (Fig. 4k), and increased PFO* labeling in NIH3T3 cells (Fig. 4l) and Hh-associated DAOY medulloblastoma cells (Fig. 4m, n). Like Hsd11β1 in ASZ cells (Fig. 3k) Retnla did not localize to DAOY cilia (Fig. 4o), but over-expression of Retnla increased clonogenic growth in a manner that was attenuated by the Hsd11β2 antagonist CNX (Fig. 4p). These data demonstrate Retnla regulates Smo-activating lipid metabolism to activate Hh signal transduction and drive cell proliferation *in vitro*.

Human iPSC-derived neuroepithelial stem (NES) cells overexpressing MYCN (NES^MYCN^) can be implanted into the cerebella of mice to model Hh-associated medulloblastoma^28^, and we found NES^MYCN^ cell express primary cilia (Fig. 5a, b). To determine if Hh target genes regulating Smo-activating lipid metabolism also regulate oncogenic Hh cell fate decisions *in vivo*, doxycycline-inducible HSD11β1 or Retnla were stably expressed in NES^MYCN^ cells (Fig. 5c). NES^MYCN^ cells were implanted into the cerebella of *Foxn1*^*nu*^ (Nu/Nu) mice. Tumor initiation and growth were monitored using non-invasive intracranial bioluminescence. HSD11β1 overexpression attenuated Hh-associated medulloblastoma tumorigenesis (3 of 12 mice, 25%) compared to Retnla overexpression (12 of 13 mice, 92%) or NES^MYCN^ control cells without overexpression of Hh target genes (24 of 25 mice, 96%) (Fig. 5d). Moreover, survival was improved in iPSC-derived NES^MYCN^ Hh-associated medulloblastomas with HSD11β1 overexpression compared to control or Retnla overexpression conditions (Fig. 5e). Histological analysis of hematoxylin and eosin-stained sections at survival endpoints showed NES^MYCN^ control medulloblastomas were comprised of hyperchromatic tumor cells with nuclear molding, indistinct to multiple small nucleoli, and indistinct cytoplasm (Fig. 5f). Abundant Homer-Wright (neuroblastic) rosettes were admixed with areas of neuropil. Tumor cells showed increased pale cytoplasm or mature neuronal phenotypes. NES^MYCN^ Hh-associated medulloblastomas with Retnla overexpression showed a similar appearance, but with fewer rosettes and increased neuropil-like stroma (Fig. 5f). HSD11β1 overexpression in NES^MYCN^ Hh-associated medulloblastoma was associated with prominent central nucleoli and vesicular chromatin, with rare rosettes and no areas of extensive neuropil-like stroma (Fig. 5f). To elucidate the gene expression programs underlying phenotypic differences across iPSC-derived NES^MYCN^ Hh-associated medulloblastomas, RNA sequencing was performed on control tumors (n = 3), tumors with HSD11β1 overexpression (n = 3), and tumors with Retnla overexpression (n = 3) (Supplementary Table 7). Differential expression and ontology analyses demonstrated enrichment in cell signaling and cell adhesion programs in medulloblastomas with overexpression of Retnla, and suppression of ciliary organization and Hh cell fate programs (e.g. endochondral ossification) in medulloblastomas with overexpression of HSD11β1 (Fig. 5g).

In summary, we define the Hh gene expression program using RNA sequencing of (1) NIH3T3 cells treated with Hh ligands (Shh, Dhh, Ihh), Smo-activating oxysterols (24(*S*),25-EC, 7β,27-DHC, 20-OHC), and SAG (Fig. 1 and Supplementary Table 1–3), (2) 10 matched pairs of tumor and adjacent skin samples from patients with newly diagnosed BCC (Fig. 2, 3b, and Supplementary Table 4), (3) 458 human medulloblastomas (Fig. 4a and Supplementary Table 5), and (4) *in vivo* genetic and iPSC models of Hh-associated medulloblastoma in mice (Fig. 4b, 5g and Supplementary Table 5, 6). Our results reveal a core Hh gene expression program comprised of 143 protein coding genes that are associated with lipid synthesis (*Hsd11β1, Retnla*), metabolism, cell signaling, cell adhesion, or angiogenesis (Supplemental Table 3). To validate our results, we integrate mechanistic and functional studies with lipidomic mass spectrometry, bacterial metabolite labeling of free sterols, and genetic and pharmacologic approaches in cells and mice. We show the Hh target gene *Hsd11β1* regulates Smo-activating lipid metabolism to inhibit Hh signal transduction and block cell proliferation *in vitro*, and that the Hh target gene *Retnla* regulates Smo-activating lipid metabolism to activate Hh signal transduction and drive cell proliferation *in vitro*. *In vivo,* Hsd11β1 attenuates Hh-associated medulloblastoma tumorigenesis and improves survival, reprogramming the cellular architecture and gene expression of the most common malignant brain tumor in children. These data provide insights into cellular mechanisms underlying ciliary and oncogenic Hh signaling and elucidate targets to treat Hh-associated cancers.

Several key questions remain regarding lipid metabolism and Hh signal transduction. First, Hsd11β1 inhibits Hh target gene expression (Fig. 3e, h) and production of Smo-activating lipids (Fig. 3j) without inhibiting Smo accumulation in primary cilia (Fig. 3d, g). Retnla activates Hh target gene expression (Fig. 4e, j), production of Smo-activating lipids (Fig. 4l, n), and Smo accumulation in primary cilia (Fig. 4d, i). These data suggest lipids may decouple Smo accumulation in primary cilia from Smo activation, as is the case for cyclopamine, a small molecule that accumulates Smo in cilia but inhibits Hh target gene expression^1–3^. Second, *Hsd11β1* expression is increased in NIH3T3 cells in response to ciliary ligands but is suppressed in Hh-associated cancers (Fig. 3b, 4b); and reminiscent of Ptch1, Hsd11β1 is both a target and a negative regulator of the Hh pathway. Thus, we propose Hsd11β1 expression represents a negative feedback loop regulating Hh signaling output that can be disrupted to drive the growth of Hh-associated cancers. Third, Hsd11β1 and Retnla regulate Hh signal transduction, at least in part, through Hsd11β2 (Fig. 3l, 4f, 4p), but the enzymes and adipokines that regulate Hh signal transduction are complex, both in terms of their subcellular localization^29^ and in terms of the structural diversity of the lipids they influence^15^. Hsd11β1 (Fig. 3k), Retnla (Fig. 4o), and Hsd11β2^12^ do not localize to cilia, but primary cilia are enriched in Smo-activating lipids^12,15^. Future investigation of mechanisms regulating the subcellular distribution of Smo-activating lipids will therefore be important for determining if Hh target genes influence the lipid composition or certain cell membranes or all cell membranes to regulate Hh signal transduction.

## Methods

This study complied with all relevant ethical regulations and was approved by the UCSF Institutional Review Board (IRB #10-01141 and #18-24633). As part of routine clinical practice, all patients who were included in this study signed a written waiver of informed consent to contribute de-identified skin and basal cell carcinoma tissue for research.

### Cell culture, treatments, and clonogenic assays

NIH3T3 cells (CRL-1658, ATCC), HEK293T cells (CRL-3216, ATCC), and DAOY cells (HTB-186, ATCC) were cultured in DMEM medium (11960069, Thermo Fisher Scientific) containing 10% fetal bovine serum (FBS) and GlutuMAX (35050079, Thermo Fisher Scientific) at 37°C in a humidified atmosphere with 5% CO_2_. ASZ001 cells (a gift from JFR) were cultured in Medium 154CF (M154CF500, Thermo Fisher Scientific) containing 3% FBS, GlutuMAX, and 0.05mM CaCl_2_. Male WTC10 iPSC cells (a gift from WAW) were differentiated into NES cells as previously described^28^. In brief, NES cells were plated on poly-L-ornithine/laminin coated wells and cultured in NES cell medium comprised of DMEM/F-12 (11-320-082, Fisher Scientific), Glutamax, N2 (400-163, Gemini Bio-Products), B27 without vitamin A (1287010, Thermo Fisher Scientific), 1.6g/L Glucose, 10–20ng/mL EGF, and 10–20ng/mL FGF at 37°C in a humidified atmosphere with 5% CO_2_.

For ciliation and Hh signaling assays, NIH3T3, ASZ, DAOY, and NES cells were transitioned to serum-free OptiMEM (51985091, Thermo Fisher Scientific) for 24 hours and treated with small molecules or appropriate vehicle controls. Lyophilized lipids were re-constituted in 100% ethanol, including 7b,27-dihydroxycholesterol-d6 (7b,27-DHC, 700137P, Avanti Polar Lipids), 24(*S*),25-epoxycholesterol (24(*S*),25-EC, 700039P, Avanti Polar Lipids), 20(*S*)-hydroxycholesterol (20(*S*)-OHC, 4474, Tocris), and 7α,27-dihydroxycholesterol-d6 (7α,27-DHC, 700136P, Avanti Polar Lipids). Cells were treated with lipid concentrations varying from 1–33 μM or ethanol as a vehicle control, as indicated in the figures and figure legends. Lyophilized recombinant Hh ligands were reconstituted in PBS (SH3002802, Cytiva) supplemented with 0.01% BSA (A2153, Sigma-Aldrich), including Sonic Hh (Shh, 1845-SH, R&D Systems), Indian Hh (Ihh, 1705-HH, R&D Systems), and Desert Hh (Dhh, 733-DH, R&D Systems). Cells were treated with Hh ligands at concentrations ranging from 0.1–25.0μg/mL or 0.01% BSA reconstituted in PBS as a vehicle control, as indicated in the figures and figure legends. Smoothened agonist (SAG, 566660, Calbiochem) was reconstituted in DMSO and cells were treated at concentrations ranging from 10–500 nM or DMSO as a vehicle control, as indicated in the figures and figure legends. Doxycycline (D9891, Sigma-Aldrich) or carbenoxolone (CNX, 30965, Tocris) were reconstituted in water and treated at concentrations of 5–10μg/mL for doxycycline and 10μM for CNX.

For clonogenic assays, 1000 cells were seeded in 10 cm^2^ plates and cultured for 10 days. Cells were fixed in methanol for 30 minutes and stained with 0.01% crystal violet (C6158, Sigma-Aldrich) for 1 hour. Plates were rinsed with H_2_O three times, air-dried, digitally scanned, and total colony area was quantified using ImageJ Colony Area^30^.

### Flow cytometry

NIH3T3 or DAOY cells were cultured in 6-well plates, detached using 0.05% Trypsin-EDTA (25300120, Thermo Scientific), and pelleted by centrifugation at 400×g for 5 minutes. Cell pellets were resuspended, washed twice with PBS, and incubated in blocking buffer comprised of PBS supplemented with 10 mg/mL BSA for 10 minutes at 4°C. Cells were washed once more before staining with 5 μg/mL Perfringolysin O*–647 reagent (PFO*, a gift from Maia Kinnebrew and Raj Rohatgi) for 30 minutes at 4°C. Cells were pelleted, washed one in PBS, and then resuspended in DMEM without serum or phenol red before analysis. Fluorescence intensity measurements were performed using a SH800 Cell Sorter (Sony) using a 638nm laser for excitation. Cells were gated based on forward scatter area (FSC-A) and height (FSC-H) using FlowJo.

### Lentiviral production and transduction

Plasmids containing constructs of interest were co-transfected into HEK293T cells with the lentiviral packaging plasmids psPAX2 (12260, Addgene) and pMD2.G (12259, Addgene). For each 10cm^2^ plate, the following DNAs were diluted into 600μL Optimem: 4.5μg construct, 6μg psPAX2, and 1.5μg pMD2.G. Separately, 36μL of TransIT-Lenti transfection reagent (MIR6606, Mirus) was diluted into 600μL of OptiMEM, briefly vortexed, and incubated at room temperature for 5 minutes. The DNA and Mirus solutions were combined and allowed to incubate at room temperature for 20 minutes. Meanwhile, the cell culture media on HEK293T cells was replaced with 9mL of pre-warmed DMEM supplemented with 1% FBS and GlutaMAX, and the transfection mixture was added dropwise onto the cells. Viral particles were harvested both 48 hours and 72 hours after transfection by passing the cell culture medium through a 0.45μM PVDF syringe filter (F5510, Denville Scientific). The filtered supernatant was used for transduction of recipient cultures, and the growth medium was supplemented with 10μg/mL polybrene (TR-1003, EMD Millipore). When larger quantities of virus were desired, all previously stated amounts were doubled to produce virus from 15cm^2^ plates.

### Epigenomic and genomic editing

NIH3T3 cells stably expressing the CRISPRi machinery dCas9-KRAB (NIH3T3^dCas9-KRAB^) were generated by producing lentivirus encoding pHR-UCOE-EF1α-dCas9-HA-2xNLS-XTEN80-KRAB-P2A-BFP (a gift from Luke Gilbert) as described above for epigenomic editing. NIH3T3 cells were transduced and sorted for the top 25% BFP expression on a SH800 Cell Sorter. Expression was validating using immunoblots for the HA epitope in the dCas9-KRAB construct as described below. To generate CRISPRi knockdowns, sgRNAs targeting promoter regions of interest were selected from optimized sgRNA sequences^31^ (Supplementary Table 8). Oligonucleotides corresponding to sgRNAs were ordered with complementary overhangs and cloned into pCRISPRia-v2 (84832, Addgene) following BstxI (R0113, New England Biolabs) and BlpI (R0585, New England Biolabs) restriction enzyme digests. Knockdown was confirmed using QPCR as described below.

To generate NIH-3T3 Smo^−/−^ cells, sgRNAs targeting early exons of Smo were selected from optimized sgRNA sequences^32^ (Supplementary Table 8), and cloned into lentiCRISPR v2 (52961, Addgene) for CRISPR/Cas9 genomic editing. Lentivirus was produced as described above and cells were transduced and selected in 2μg/mL puromycin for 2–3 days or until a control population of cells without a puromycin resistance cassette died. Monoclonal cells were expanded in 96-well plates and knockout was confirmed using immunofluorescence for ciliary Smo, or immunoblots for total Smo, as described below.

### Immunoblotting

Cells were detached using 0.05% trypsin-EDTA (25300120, Thermo Fisher Scientific) and pelleted at 300xg for 5 minutes. Cell pellets were resuspended in Dulbecco’s phosphate buffered saline (DPBS, SH3002802, GE Healthcare) and re-pelleted at 300×g for 5 minutes. Lysates were prepared by re-suspending cell pellets in lysis buffer comprised of 1% NP-40, 150mM NaCl, 50mM Tris pH 7.4, protease inhibitor (539134, EMD Millipore), and phosphatase inhibitor (524627, EMD Millipore) in 4°C DPBS. Lysates were centrifuged at 22,000×g for 15 minutes at 4°C, and the supernatant was retained for immunblotting. Protein concentrations were determined using a Bradford assay (5000002, BioRad). Proteins were separated on pre-cast sodium dodecyl sulfate-polyacrylamide 4–20% gels (4561093 or 4561096, BioRad), transferred to nitrocellulose membranes (1620094, Bio-Rad), blocked with 5% milk in Tris-buffered saline (10mM Tris, pH 8.0, and 150mM NaCl) with 0.1% Tween (TBS-T) for 1 hour at room temperature, and incubated with primary antibodies for 1 hour in blocking solution. Primary antibodies included anti-FLAG (1:1000, Rabbit, 14793, Cell Signaling Technology), anti-GAPDH (1:1000, Mouse, MA515738, Thermo Fisher Scientific), anti-HA (1:1000, Mouse, 2999, Cell Signaling Technology), or anti-Smo (1:1000, 20787, Proteintech). Membranes were washed with TBS-T and incubated with secondary antibodies for 1 hour in blocking solution. Secondary antibodies included Peroxidase AffiniPure Goat Anti-Rabbit IgG (1:5000, Goat, 111-035-144, Jackson ImmunoResearch) or Peroxidase AffiniPure Goat Anti-Mouse IgG (1:5000, Goat, 111-035-146, Jackson ImmunoResearch). Membranes were developed using SuperSignal West Pico or Femto PLUS Chemiluminescent Substrate (34577 or 34096, ThermoFisher Scientific) on autoradiography film (A8815, MTC Bio).

### Immunofluorescence and confocal microscopy

Immunofluorescence for NIH3T3, DAOY, ASZ, and NES cells was performed on glass coverslips. Cells were fixed in 4% paraformaldehyde for 7 minutes at room temperature. All antibody incubations were performed in blocking solution (10% BSA, 0.1% Triton X-100) for 1 hour at room temperature or overnight at 4°C. Primary and secondary antibodies included anti-Arl13b (1:1000, Rabbit, Arl13b, Proteintech), anti-Smo (1:1000, Rabbit, ab72130, Abcam), anti-FLAG-M2 (1:1000, Mouse, F1804, Sigma-Aldrich), donkey anti-mouse linked to Alexa Fluor 488 (1:2000, A21202, Invitrogen), or donkey anti-rabbit linked to Alexa Fluor 568 (1:2000, A10042, Invitrogen). DNA was stained using Hoescht 33342 (1:5000, H3570, Life Technologies) and coverslips were mounted in ProLong Diamond Antifade Mountant (P36965, Thermo Fisher Scientific).

Fluorescence microscopy was performed on a LSM 800 confocal laser scanning microscope (Zeiss). Acquisition parameters were controlled using Zeiss Zen v2.3. Images were collected at room temperature (25°C) using a Plan-APOCHROMATIC 63x/1.4 oil immersion objective.

Ciliary Smo intensity was quantified from 2D maximum projections of 16-bit TIFF images, and Smo signal (SS) was measured by tracing the cilia using Arl13b as a marker in ImageJ. Background was subtracted by averaging the equivalent measurement in the regions immediately adjacent to each cilium (AL and AR) for a final formula of (SS – (AL + AR)/2). Ciliary length was also measured in ImageJ, and ciliary prevalence was quantified as the percentage of nucleated cells expressing primary cilia.

Staining with PFO*–647 for immunofluorescence, as opposed to flow cytometry as described above, was performed using cells that were seeded onto glass coverslips and blocked in 4°C PBS supplemented with 10mg/mL BSA for 15 minutes. PFO*–647 was diluted in blocking buffer (1:45) and incubated with cells for 30 minutes followed by one wash in PBS with Hoescht 33342 (1:1000). Cells were fixed in 4% paraformaldehyde diluted in PBS (15710, Electron Microscopy) for 7 minutes, washed with PBS, and coverslips were mounted in ProLong Diamond Antifade Mountant. PFO*–647 signal intensity was quantified from 2D maximum projections of 16-bit TIFF images, and regions of interest were drawn around clusters comprised of 10 cells in ImageJ. PFO*–647 integrated density was divided by the measurement area to give a signal/area ratio. Data from experimental cells were normalized to control cells.

### Quantitative polymerase chain reaction

RNA was isolated from cells using the RNeasy Mini Kit (74106, Qiagen) and Qiacubes (Qiagen). cDNA was synthesized using the iScript cDNA synthesis kit (1708891BUN, BioRad), and genes of interest were amplified using PowerUp SYBR Green Master Mix (A257342, Thermo Fisher Scientific) on a Life Technologies QuantStudio 6 Flex Real Time PCR System (4485694, ThermoFisher Scientific). The ddCt method relative to *Gapdh* expression was used to quantify gene expression from primers that were selected from PrimerBank^33^ (Supplementary Table 8).

### RNA sequencing and analysis

Triplicate cultures of NIH3T3 wildtype or NIH3T3 Smo^−/−^ cells were treated with vehicle controls (DMSO, H_2_O, ethanol), Hh ligands (Shh, Ihh, Dhh), Hh-activating oxysterols (7b,27-DHC, 24(*S*)-25-EC, 20(*S*)-OHC), a non-Hh activating oxysterol with related structure (7α,27-DHC), or SAG. Across 2 cell lines, 6 conditions, and biological triplicates, this yielded a total of 72 individual NIH3T3 cultures that were analyzed using RNA sequencing. RNA was isolated from cultured cells as described above for QPCR but was prepared for RNA sequencing using the Illumina TruSeq stranded mRNA kit (20020594, Illumina). Briefly, this kit enriches for mRNA using poly-T beads and ligates sequencing adaptors to cDNA for next generation sequencing. Samples were sequenced on an Illumina NovaSeq with a depth of least 25 million 100 bp paired-end reads per sample. Sequencing reads were processed using Trimmomatic^34^ to remove leading and trailing bases with quality scores below 28 as well as bases that did not have an average quality score of 28 within a sliding window of 4 bases. Any reads shorter than 75 bases after trimming were removed. Reads were subsequently mapped to the mouse reference genome GRCm38.p6 using HISAT2 with default parameters^35,36^, resulting in ~90% of reads mapping to annotated genes for each sample. For differential expression analysis, exon level count data were extracted from mapped HISAT2 data using featureCounts^37^. Differential expression analysis was performed in R using DESeq2^38^ with the ‘apeglm’ parameter to calculate log fold changes after setting a false discovery rate of 0.05^38^. Differentially expressed genes were identified as those with log_2_ fold changes ≥1 and adjusted p-value ≤0.05 (Supplementary Table 1–3).

Newly diagnosed BCC and matched normal skin samples were isolated during Mohs surgeries from 10 patients (a gift from Sarah Arron). Tissue samples were mechanically lysed using a TissueLyser II (Qiagen) according to the manufacturer’s instructions. DNA and RNA were extracted from lysed tissue using the AllPrep DNA/RNA/miRNA Universal Kit (80224, Qiagen). Library preparation was performed using the TruSeq Stranded Total RNA Library Prep Gold Kit (20020598, Illumina) and 150 bp paired-end reads were sequenced on an Illumina NovaSeq 6000 to at least 88 million reads per sample. Quality control of FASTQ files was performed with FASTQC (http://www.bioinformatics.babraham.ac.uk/projects/fastqc/). Reads were trimmed with Trimmomatic to remove leading and trailing bases with quality scores below 28, and any bases that did not have an average quality score of 28 within a sliding window of 4 bases. Any reads shorter than 110 bases after trimming were removed. Reads were mapped to the human reference genome GRCh38 using HISAT2 with default parameters. For downstream expression analysis, extracted exon level count data were extracted from mapped HISAT2 output using featureCounts. Differential expression analysis was performed in R with DESeq2, using the ‘apeglm’ parameter to accurately calculate log fold changes and setting a false discovery rate of 0.05. Differentially expressed genes were identified as those with log_2_ fold changes ≥1 and adjusted p-value ≤0.05 (Supplementary Table 4).

RNA sequencing and analysis of human Hh-associated medulloblastomas (n=250) compared to human Group 3 medulloblastomas (n=208) were previously described^21–23^ (Supplementary Table 5). RNA sequencing and analysis of Hh-associated medulloblastomas from the *Math1-Cre SmoM2*^*c*^ genetically engineered mouse models (n=3) or *SmoM2*^*c*^ littermate control cerebella (n=3) were previously described^17^ (Supplementary Table 6).

RNA sequencing and analysis of iPSC-derived NES^MYCN^ Hh-associated medulloblastomas was performed as described above. Tumors were isolated from mice by dissection, and mechanically lysed using a TissueLyser II (Qiagen) according to the manufacturer’s instruction. RNA was isolated as described above for QPCR, and was similarly prepared for RNA sequencing using the Illumina TruSeq stranded mRNA kit. Samples were sequenced on an Illumina NovaSeq with a depth of least 25 million 100 bp paired-end reads per sample. Sequencing reads were processed using Trimmomatic^34^ to remove leading and trailing bases with quality scores below 28 as well as bases that did not have an average quality score of 28 within a sliding window of 4 bases. Any reads shorter than 75 bases after trimming were removed. Reads were subsequently mapped to the mouse reference genome GRCh38.p6 using HISAT2 with default parameters^35,36^, resulting in ~90% of reads mapping to annotated genes for each sample. Differential expression analysis was performed in R with DESeq2, using the ‘apeglm’ parameter to accurately calculate log fold changes and setting a false discovery rate of 0.05. Differentially expressed genes were identified as those with log_2_ fold changes ≥1 and adjusted p-value ≤0.05 (Supplementary Table 7).

Pathway analyses for basal cell carcinoma and NES medulloblastoma data set were performed using Gene Set Enrichment Analysis (GSEA) with preranking to determine if differentially expressed genes belonged to common biological pathways^39^. Gene rank scores were calculated using the formula: sign(log2FC) × −log10(P). Pathways were defined using Human_GOBP_AllPathways_no_GO_iea_December_01_2022_symbol.gmt, a gene set file that is regularly maintained and updated by the Bader laboratory. Positive and negative enrichment files were obtained by carrying out 2000 permutations. A pathway enrichment map was generated using EnrichmentMap in Cytoscape to visualize pathway analysis results. Nodes with FDR q-value ≤0.05, p-value ≤0.05, and nodes sharing gene overlaps with Jaccard + Overlap Combined (JOC) threshold of 0.375 were connected by blue lines (edges) to generate network maps. Clusters of related pathways were identified and annotated using AutoAnnotate in Cytoscape, which relies on a Markov Cluster algorithm that connects pathways by shared keywords in the description of each pathway. The resulting groups of pathways were designated as consensus pathways that are annotated in circles in the figures.

### Lipidomic mass spectrometry

Sterol and oxysterol quantitation was carried out by utra-high performance liquid chromatography-tandem mass spectrometry (UPLC-MS/MS) using stable isotope-labelled internal standards (d_7_-cholesterol, d7-7-dehydrocholesterol, ^13^C3-desmosterol, and ^13^C3-lanosterol for sterols and d7-7-ketocholesterol for oxysterols) as previously described^12,40^.

### Mouse xenografts

Immunocompromised Nu/Nu 6–8-week old female mice (002019, The Jackson Laboratory) were used for *in vivo* experiments that were performed in the UCSF Helen Diller Animal Facility. All experiments were performed in accordance with institutional policy and with approval from UCSF IACUC (AN191840). Differentiated NES cells were transduced with a pCDH-CAG-3xFLAG-MYCN-mScarlet-Luciferase lentiviral construct (NES^MYCN^). NES^MYCN^ cells were transduced with either pLV-TreGS-Hsd11b1-FLAG or pLV-TreGS-Retnla-FLAG and selected using 2μg/mL puromycin. Construct expression was induced with doxycycline *in vitro* at 2.5μg/mL and *in vivo* at 100μg/mL (Sigma-Aldrich, D9891). For *in vivo* induction, doxycycline water was changed every 2–3 days. Cerebellar NES medulloblastomas were generated by injecting 300,000 cells in 5μL of NES cell medium using stereotactic surgical equipment with the following coordinates: lambda 2mm right, 2mm down, and 2mm deep. Tumor growth was monitored with bioluminescence imaging on an IVIS Spectrum imager (PerkinElmer). For BLI monitoring, mice were injected with 100μL of 30mg/mL D-luciferin diluted in sterile water (LUCK, Goldbio) and imaged after 15 minutes. Quantification of bioluminescence readings were performed using LivingImage. Mice were euthanized at pre-determined humane endpoints for intracranial tumor growth, such as cachexia, head tilt, or respiratory distress.

### Histology and light microscopy

For iPSC-derived NES^MYCN^ Hh-associated medulloblastomas, deparaffinization and rehydration of 5μm formalin-fixed, paraffin-embedded (FFPE) tissue sections and H&E staining were performed using standard procedures. All histological experiments were imaged on a BX43 light microscope (Olympus) and analyzed using the Olympus cellSens Standard Imaging Software package.

### Statistics

All experiments were performed with independent biological replicates and repeated, and statistics were derived from biological replicates. Biological replicates are indicated in each figure panel or figure legend. No statistical methods were used to predetermine sample sizes, but sample sizes in this study are similar or larger to those reported in previous publications. Data distribution was assumed to be normal, but this was not formally tested. Investigators were blinded to conditions during clinical data collection and analysis of mechanistic or functional studies. Bioinformatic analyses were performed blind to clinical features, outcomes, or molecular characteristics. The clinical samples used in this study were retrospective and nonrandomized with no intervention, and all samples were interrogated equally. Thus, controlling for covariates among clinical samples was not relevant. Cells and animals were randomized to experimental conditions. No clinical, molecular, or cellular data points were excluded from the analyses. Lines represent means, and error bars represent standard error of the means. Results were compared using Student’s t-tests, which are indicated in figure panels or figure legends alongside approaches used to adjust for multiple comparisons. In general, statistical significance is shown using asterisks (*p≤0.05, **p≤0.01, ***p≤0.0001), but exact p-values are provided in figure panels or figure legends when possible.

## Figures and Tables

**Figure 1:**
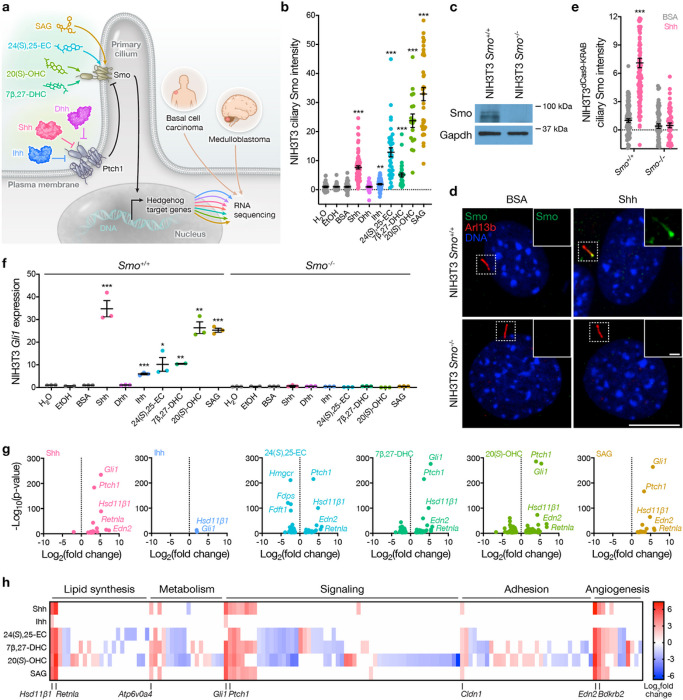
The Hedgehog gene expression program. **a**, Experimental design and workflow to define the Hh gene expression program in response to ciliary ligands and in the context of oncogenic Hh signaling. **b**, Quantitative immunofluorescence microscopy for ciliary Smo in NIH3T3 cells after stimulation with 1ug/mL Shh, Dhh, or Ihh, 33μM 24(S)-25-EC, 7β,27-DHC, or 20(S)-OHC, or 50nM SAG. **c**, Immunoblot assessment of Smo in NIH3T3 wildtype versus *Smo*^−/−^ cells. **d**, Immunofluorescence microscopy for Smo (green), primary cilia marked by Arl13b (red), or DNA (blue) in NIH3T3 wildtype versus *Smo*^−/−^ cells without or without Hh pathway stimulation with 1μg/mL Shh. Scale bars, 10μm and 1μm. **e**, Quantitative immunofluorescence microscopy for ciliary Hh in NIH3T3 wildtype versus *Smo*^−/−^ cells with or without Hh stimulation as in **d. f**, QPCR assessment of the Hh target gene *Gli1* in NIH3T3 wildtype versus *Smo*^−/−^ cells with or without Hh stimulation as in **b. g**, Volcano plots showing differentially expressed genes from RNA sequencing of NIH3T3 cells treated with versus without Hh stimulation as in b and f. Differentially expressed genes were defined by log_2_ fold change ≥1 and FDR ≤0.05 compared to appropriate vehicle controls (H_2_O, BSA, EtOH) after subtraction of any genes meeting these criteria from RNA sequencing of NIH3T3 *Smo*^−/−^ cells treated with the same ligands, and (for oxysterol conditions) subtraction of any genes meeting these criteria from RNA sequencing of NIH3T3 cells treated with 7α,27-DHC, an oxysterol does not bind to Smo nor activate the Hh pathway. Conserved genes of interest across ligand conditions are annotated. No differentially expressed genes were identified after NIH3T3 treatment with Dhh. **h**, Heat map showing 143 differentially expressed protein coding genes from **g** organized by manually curated gene ontologies. Conserved genes of interest that were enriched after Hh pathway activation are annotated. Lines represent means and error bars represent standard error of the means. Student’s t-tests, *p≤0.05, **p≤0.01, ***p≤0.0001.

**Figure 2:**
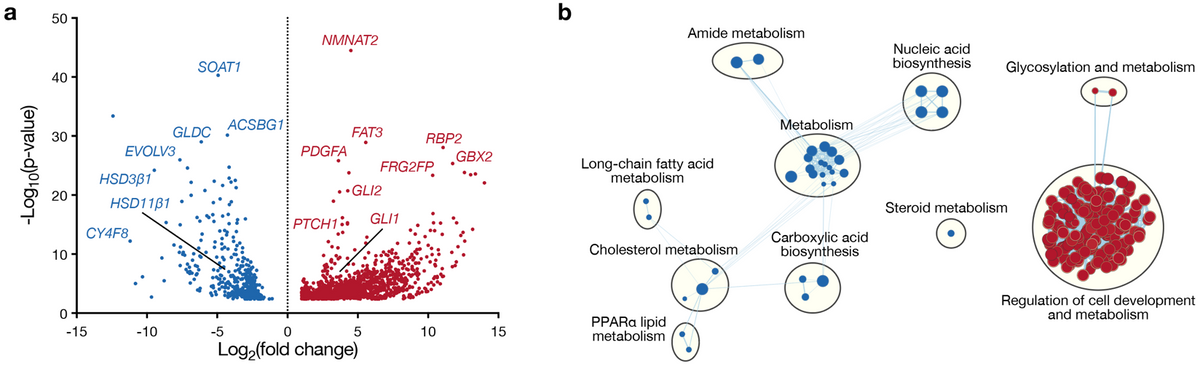
The Hedgehog gene expression program regulates lipid metabolism in basal cell carcinoma. **a,** Volcano plot showing differentially expressed genes from RNA sequencing of newly diagnosed human basal cell carcinoma (n=10) compared to matched paired adjacent skin samples (n=10). **b,** Network of gene circuits distinguishing newly diagnosed human basal cell carcinoma (n=10) from matched paired adjacent skin samples (n=10) using RNA sequencing. Nodes represent pathways and edges represent shared genes between pathways (p≤0.05, FDR≤0.05). Red nodes are enriched and blue nodes are suppressed in basal cell carcinoma compared to adjacent skin.

**Figure 3:**
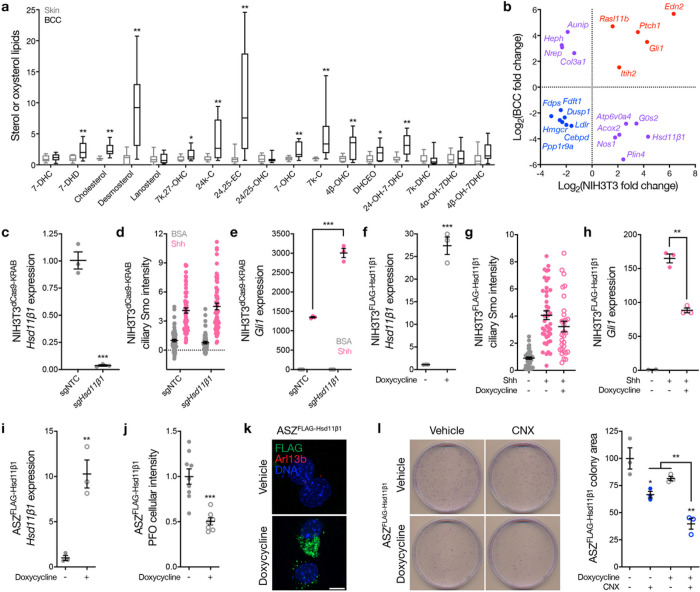
The Hedgehog target gene *Hsd11β1* regulates lipid metabolism to inhibit Hedgehog signaling. **a,** Lipidomic mass spectrometry quantitation of oxysterols from 10 human basal cell carcinomas (BCC) compared to matched normal skin samples from the same patients. Lines represent means and boxes represent upper and lower quartiles. Whiskers show min to max values. **b,** Differentially expressed genes with log_2_ fold change ≥1 intersecting between human BCCs (Fig. 2 and Supplementary Table 4) and NIH3T3 cells (Fig. 1 and Supplementary Table 3). **c**, QPCR assessment of *Hsd11β1* in NIH3T3^dCas9-KRAB^ cells transduced with an sgRNA targeting *Hsd11β1* (*sgHsd11β1*) or a non-targeted control sgRNA (sgNTC). **d**, Quantitative immunofluorescence microscopy for ciliary Smo in NIH3T3^dCas9-KRAB^ cells ± *Hsd11β1* suppression ± 1μg/mL SHH showing no differences across conditions. **e**, QPCR assessment of the Hh target gene *Gli1* in NIH3T3^dCas9-KRAB^ cells with genetic and pharmacologic perturbations as in **d. f**, QPCR assessment of *Hsd11β1* in NIH3T3^FLAG-Hsd11β1^ cells ± doxycycline induction of Hsd11β1 overexpression. **g**, Quantitative immunofluorescence microscopy for ciliary Smo in NIH3T3^FLAG-Hsd11β1^ cells ± doxycycline-induced Hsd11β1 overexpression ± 1μg/mL SHH showing no differences across conditions. **h**, QPCR assessment of *Gli1* in NIH3T3^FLAG-Hsd11β1^ cells with genetic and pharmacologic perturbations as in **g. i**, QPCR assessment of *Hsd11β1* in NIH3T3^FLAG-Hsd11β1^ cells ± doxycycline-induced Hsd11β1 overexpression. **j**, PFO*–647 free sterol staining and quantitative microscopy in ASZ^FLAG-Hsd11β1^ cells ± doxycycline-induced Hsd11b1 overexpression. **k**, Immunofluorescence microscopy for Smo (green), primary cilia marked by Arl13b (red), or DNA (blue) in ASZ^FLAG-Hsd11β1^ cells ± doxycycline induction of Hsd11b1 overexpression. Scale bar, 10μm. **l**, ASZ^FLAG-Hsd11β1^ clonogenic growth ± 10μM carbenoxolone (CNX) to inhibit Hsd11β2 ± doxycycline-induced Hsd11β1 overexpression. Lines represent means and error bars represent standard error of the means. Student’s t-tests, *p≤0.05, **p≤0.01, ***p≤0.0001.

**Figure 4:**
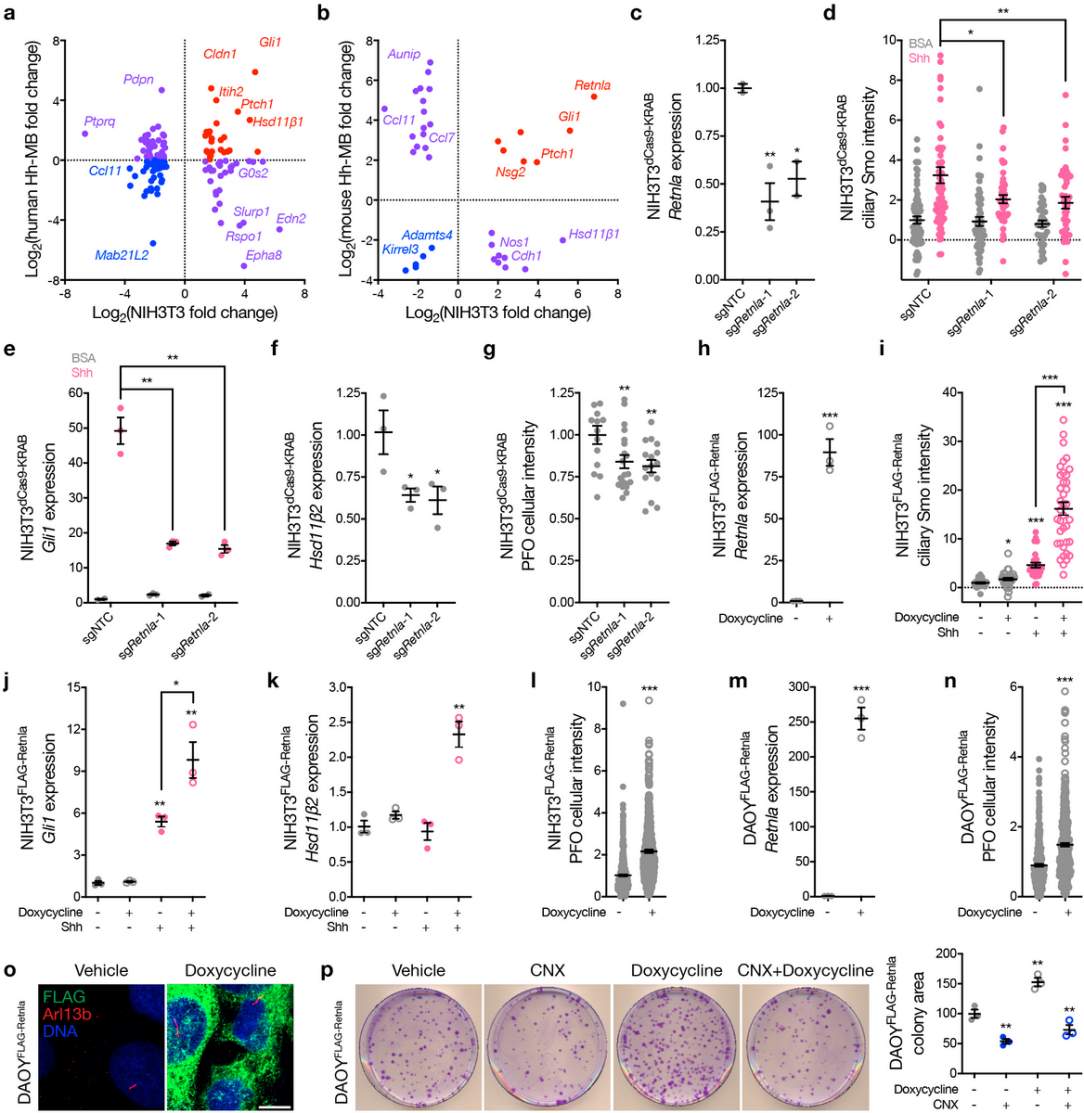
The Hedgehog target gene *Retnla* regulates lipid metabolism to activate Hedgehog signaling. **a,** Differentially expressed genes with log_2_ fold change ≥1 intersecting between human Hh-associated medulloblastomas (Supplementary Table 5) and NIH3T3 cells (Fig. 1 and Supplementary Table 3). **b**, Differentially expressed genes with log_2_ fold change ≥1 intersecting between mouse Hh-associated medulloblastomas (Supplementary Table 6) and NIH3T3 cells (Fig. 1 and Supplementary Table 3). **c**, QPCR assessment of *Retnla* in NIH3T3^dCas9-KRAB^ cells transduced with 2 independent sgRNAs targeting *Retnla* (*sgRetnla*) or a non-targeted control sgRNA (sgNTC). **d**, Quantitative immunofluorescence microscopy for ciliary Smo in NIH3T3^dCas9-KRAB^ cells ± Retnla suppression ± 1μg/mL SHH. **e**, QPCR assessment of the Hh target gene *Gli1* in NIH3T3^dCas9-KRAB^ cells with genetic and pharmacologic perturbations as in d. f, QPCR assessment of *Hsd11β2* in NIH3T3^dCas9-KRAB^ cells with genetic and pharmacologic perturbations as in **d. g**, PFO*–647 free sterol staining and quantitative microscopy in NIH3T3^dCas9-KRAB^ cells ± *Retnla* suppression. **h**, QPCR assessment of *Retnla* in NIH3T3^FLAG-Retnla^ cells ± doxycycline induction of Retnla overexpression. **i**, Quantitative immunofluorescence microscopy for ciliary Smo in NIH3T3^FLAG-Retnla^ cells ± doxycycline-induced Retnla overexpression ± 1μg/mL SHH. **j**, QPCR assessment of *Gli1* in NIH3T3^FLAG-Hsd11b1^ cells with genetic and pharmacologic perturbations as in **i. k,** QPCR assessment of *Hsd11β2* in NIH3T3^FLAG-Hsd11b1^ cells with genetic and pharmacologic perturbations as in **i. l**, PFO*–647 free sterol staining and flow cytometry quantification in NIH3T3^FLAG-^Retnla cells ± doxycycline-induced Retnla overexpression. **m**, QPCR assessment of *Retnla* in DAOY^FLAG-^Retnla cells ± doxycycline-induced Retnla overexpression. **n**, PFO*–647 free sterol staining and flow cytometry quantification in DAOY^FLAG-Retnla^ cells ± doxycycline-induced Retnla overexpression. **o,** Immunofluorescence microscopy for Smo (green), primary cilia marked by Arl13b (red), or DNA (blue) in DAOY^FLAG-Retnla^ cells ± doxycycline-induced Retnla overexpression. Scale bar, 10μm. **p**, DAOY^FLAG-Retnla^ clonogenic growth ± 10μM carbenoxolone (CNX) to inhibit Hsd11β2 ± doxycycline-induced Retnla overexpression. Lines represent means and error bars represent standard error of the means. Student’s t-tests, *p≤0.05, **p≤0.01, ***p≤0.0001.

**Figure 5:**
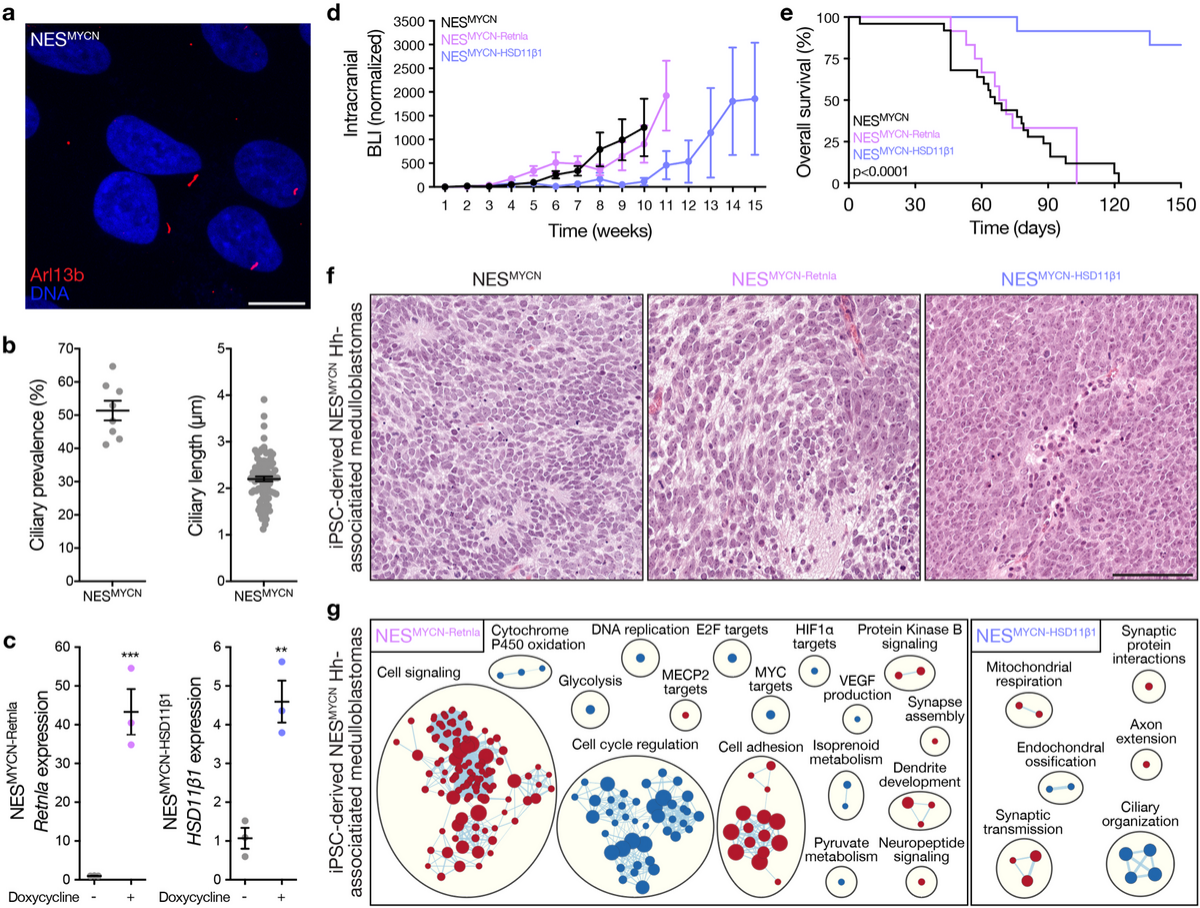
Hedgehog target genes regulating lipid metabolism control the growth of Hedgehog-associated medulloblastoma. **a**, Immunofluorescence microscopy showing primary cilia marked by Arl13b (red) or DNA (blue) in human iPSC-derived NES^MYCN^ cells. Scale bar, 10μm. **b**, Quantification immunofluorescence microscopy of ciliary prevalence (left) or ciliary length (right) in NES^MYCN^ cells. **c**, QPCR assessment of *Retnla* or *HSD11β1* in NES^MYCN^ cells ± doxycycline-induced overexpression. Lines represent means and error bars represent standard error of the means. Student’s t-tests, *p≤0.05, **p≤0.01, ***p≤0.0001. **d**, Intracranial bioluminescence (BLI) measurements from NES^MYCN^ (n=25), NES^MYCN-HSD11β1^ (n=12), or NES^MYCN-Retnla^ (n=13) Hh-associated medulloblastomas in Nu/Nu mice. Lines represent means and error bars represent standard error of the means. **e**, NES^MYCN^ Hh-associated medulloblastoma survival. Number of mice per condition as in d. Log-rank test. **f**, Hematoxylin and eosin-stained sections at survival endpoints for NES^MYCN^ Hh-associated medulloblastomas showing differences in rosette formation and neuropil-like stroma across conditions. Scale bar, 100μm. **g**, Network of gene circuits distinguishing NES^MYCN-Retnla^ medulloblastomas (n=3) or NES^MYCN-HSD11β1^ medulloblastomas (n=3) from NES^MYCN^ medulloblastomas (n=3) using RNA sequencing. Nodes represent pathways and edges represent shared genes between pathways (p≤0.05, FDR≤0.05). Red nodes are enriched and blue nodes are suppressed.

## Data Availability

RNA sequencing of NIH3T3 cultures (n=72), human basal cell carcinomas with paired normal skin samples (n=20), and NES Hh-associated medulloblastomas (n=9) have been deposited to the NCBI Gene Expression Omnibus under accession GSE233744 (https://www.ncbi.nlm.nih.gov/geo/query/acc.cgi?acc=GSE233744). RNA sequencing of mouse or human Hh-associated medulloblastomas and control samples were previously deposited to the NCBI Gene Expression Omnibus under accession GSE104633 (https://www.ncbi.nlm.nih.gov/geo/query/acc.cgi?acc=GSE104633) and to the European Genome-Phenome Archive under accession EGAD00001008458 (https://blog.ega-archive.org/datasets/EGAD00001008458), EGAD00001006305 (https://ega-archive.org/datasets/EGAD00001006305), and EGAD00001004435 https://ega-archive.org/datasets/EGAD00001004435). The publicly available datasets GRCh38 (hg38, https://www.ncbi.nlm.nih.gov/assembly/GCF_000001405.39/) and GRCm38 (m38, https://www.ncbi.nlm.nih.gov/assembly/GCF_000001635.26) were used in this study.
